# Novel simulator of endoscopic hemostasis with actual endoscope and devices

**DOI:** 10.1016/j.vgie.2022.10.004

**Published:** 2022-11-29

**Authors:** Takeshi Kanno, Yutaro Arata, Yutaka Hatayama, Tomoyuki Koike, Atsushi Masamune

**Affiliations:** 1 Division of Gastroenterology, Tohoku University Graduate School of Medicine, Sendai, Japan; 2 Department of Education and Support for Regional Medicine, Tohoku University Hospital, Sendai, Japan; 3 Graduate Medical Education center, Tohoku University Hospital, Sendai, Japan

**Keywords:** 3D, 3-dimensional, CT, computed tomography GI, gastrointestinal

## Abstract

Video 1 Demonstration of novel simulator of endoscopic hemostasis.

Demonstration of novel simulator of endoscopic hemostasis.

Endoscopic hemostasis is an essential skill for endoscopists and has been the first-line treatment.^1^ For example, we reported that 70% of bleeding ulcers were treated with endoscopic hemostasis.^2^ Endoscopic hemostasis, similar to most techniques, is currently acquired through on-the-job training with real patients. However, such high-risk situations are not preferable for trainees. To perform a hemostatic procedure safely, the operator needs skills such as maintaining an appropriate view, stabilizing the scope, and controlling hemostatic devices precisely.

Hemostatic clips and a coagulation grasper are common devices for endoscopic hemostasis, whether mechanical or thermocoagulation, and both are deemed effective for definitive hemostasis.^3^ Although several virtual reality simulator trainings have been proposed, these machines are very expensive, and trainees are unable to use actual hemostatic devices.^4^ While there have been reports that endoscopic techniques have been improved by ex vivo simulators that resembled Dieulafoy lesions with vessels sewn into an explanted porcine stomach,^5^ this model may not be routinely accessible to beginner trainees. Therefore, we developed a novel GI bleeding simulator to practice endoscopic hemostasis by using hemostatic clips or a hemostatic grasper.

The GI bleeding model comprises a disposable hemorrhagic ulcer and reusable stomach lumen. The ulcer is made of elastic ethylene resin with several vessels on the surface; thus, spurt bleeding resembling Forrest classification subtype (Ia) can be reproduced manually (Fig. 1).

**Figure 1:**
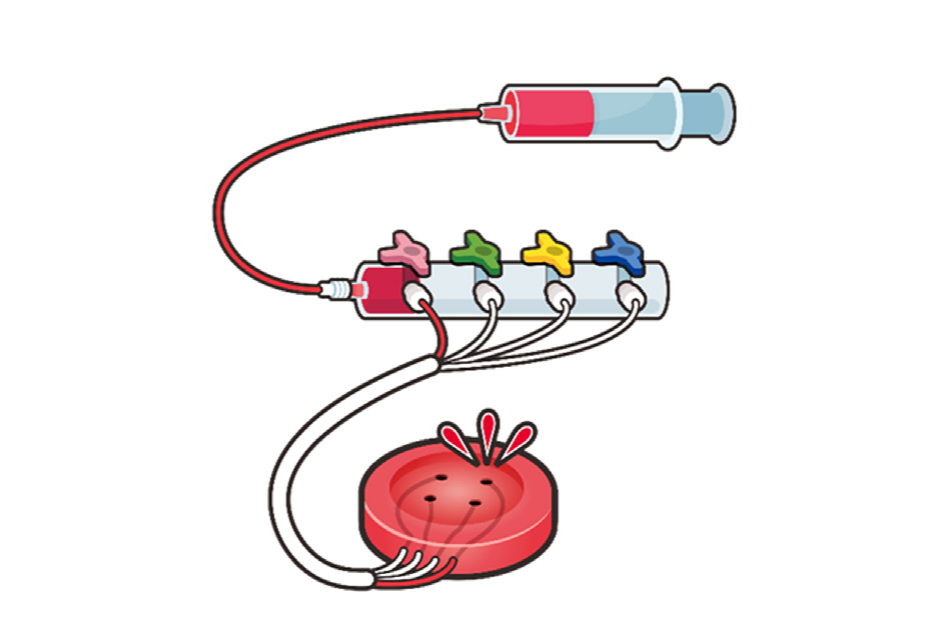
Schema of the GI bleeding ulcer model.

The bleeding ulcer model for the hemostatic grasper is equipped with electrodes beneath the ulcer (Fig. 2) to avoid electric shock when using monopolar forceps; the user can peel off the sticker on its back and install it anywhere from the stomach to the duodenal bulb (Fig. 3). The potential advantage of this simulator over the ex vivo model for hemostasis training is the ease of setup in not having to prepare the porcine model and insert vessels.

**Figure 2:**
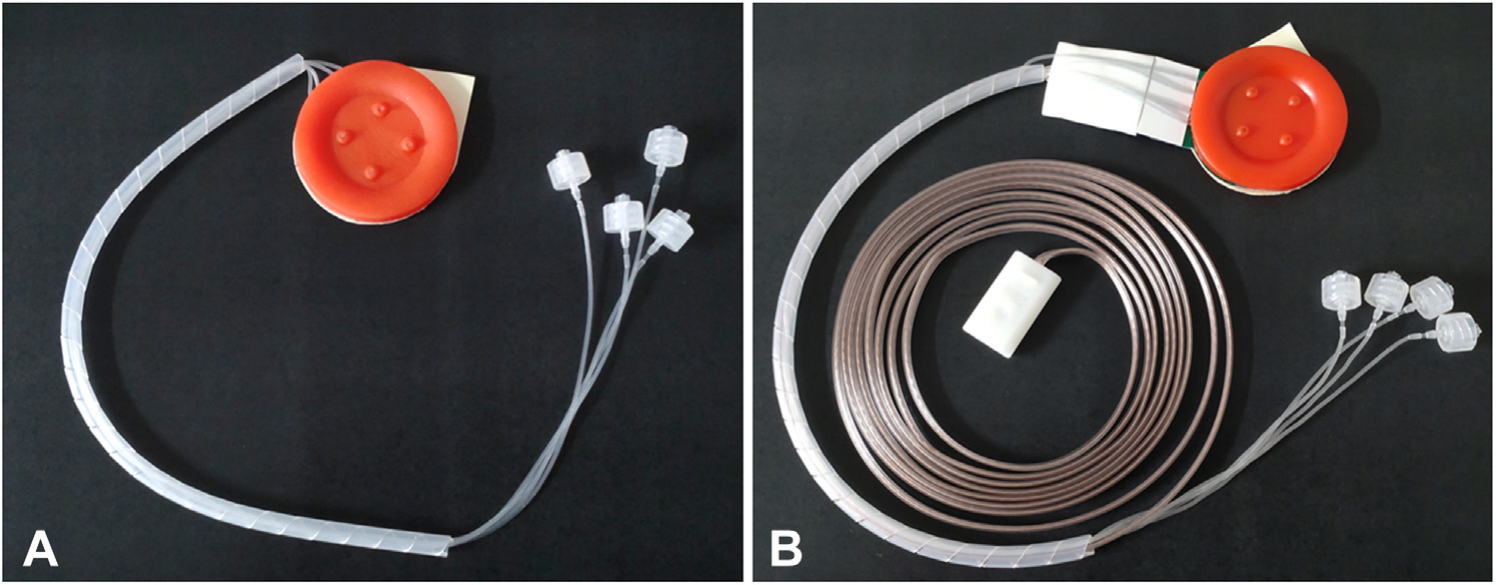
**A,** The ulcer model for hemostatic clips; **B,** The ulcer model with electrodes and electric wiring for hemostatic grasper.

**Figure 3:**
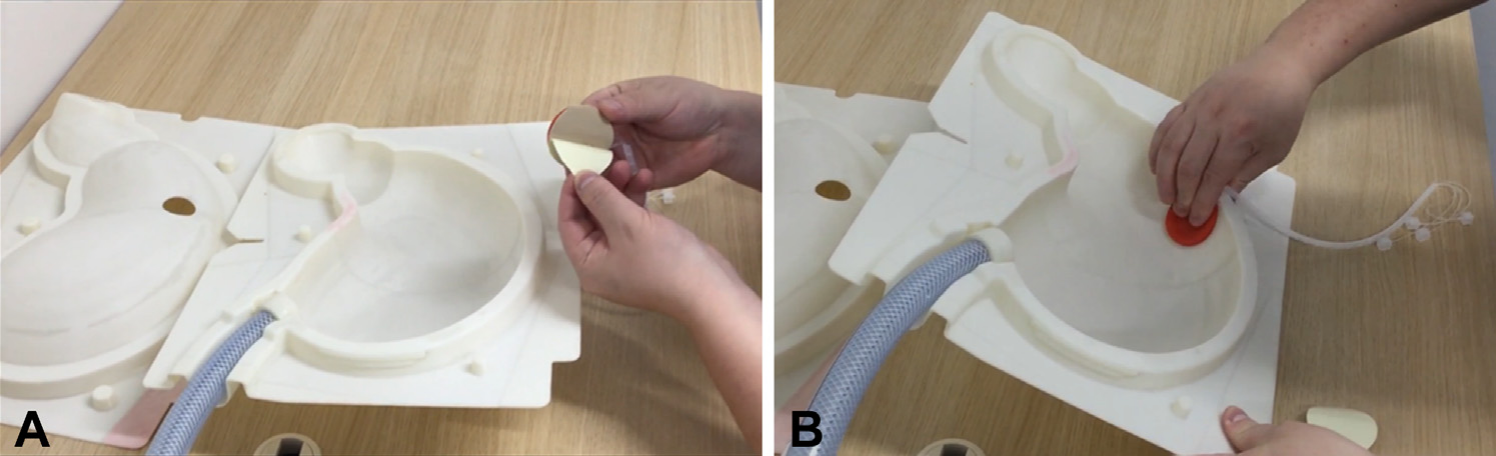
**A,** Peeling and **B,** attaching of the ulcer model to the lumen side.

The stomach lumen is made of styrene resin and designed based on 3-dimensional data from a human CT scan. This model was attached to the pharyngo-esophageal part (Fig. 4).

**Figure 4:**
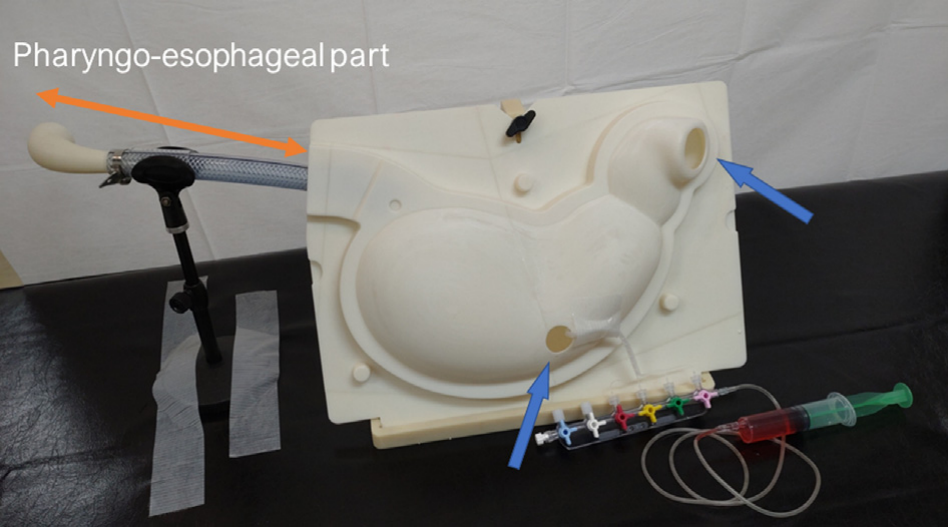
The pharyngo-esophageal part connecting to the gastric lumen (*orange arrow*). To avoid interference with endoscopic operability, the wiring was removed through holes located on the posterior wall of the stomach and the distal side of the duodenal lumen (*blue arrows*).

The reusable stomach lumen is water- and impact-resistant and can allow passage of an actual endoscope (Fig. 5). Difficulty in the procedure can be adjusted by changing the location of the ulcer (Figs. 6 and 7; Video 1, available online at www.giejournal.org). For example, when an ulcer is placed on the greater curvature of the antral area in the stomach, the endoscope can be easily stabilized, and hemostasis can be easily achieved if the hemostatic clips can properly grasp the bleeding vessel. However, in cases where procedures are performed using a retroflex approach, such as the posterior wall of the upper body of the stomach, maintenance of an appropriate distance between the endoscope and the lesion is not easy, even for experts. Reproduction of such high-difficulty lesions can help advanced endoscopists to maintain and improve their techniques.

**Figure 5:**
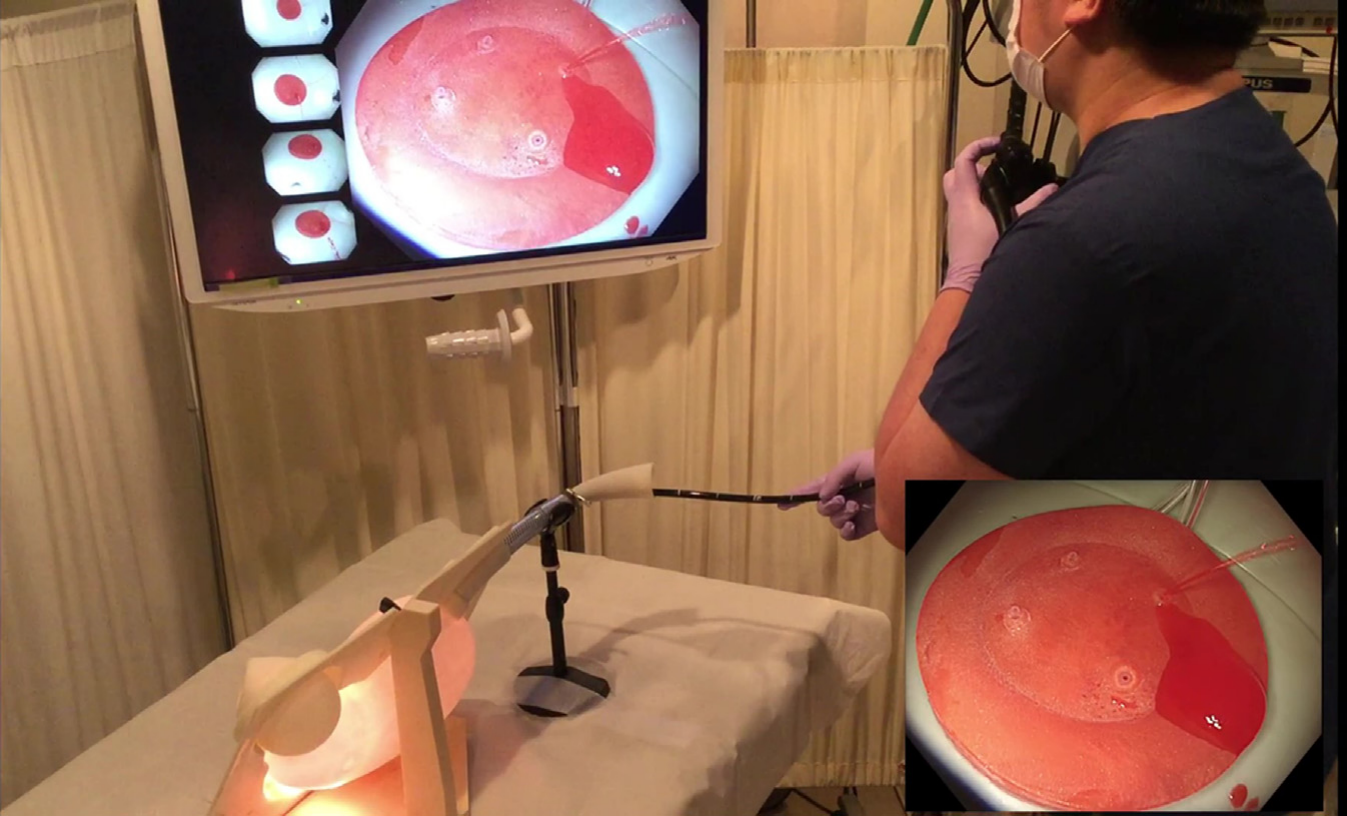
Confirming bleeding from the ulcer model using an actual endoscope.

**Figure 6:**
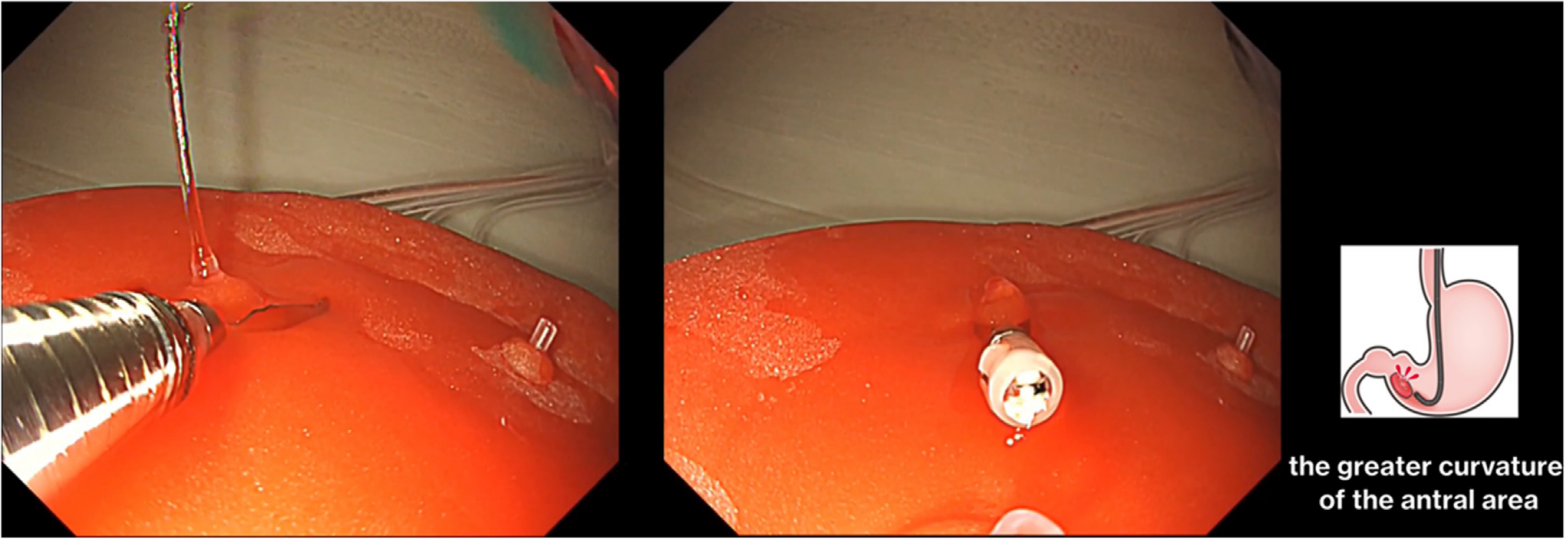
Bleeding ulcer at the greater curvature of the antral area, with hemostasis easily achieved due to better stabilization of the endoscope, allowing for the hemostatic clips to properly grasp the bleeding vessel.

**Figure 7:**
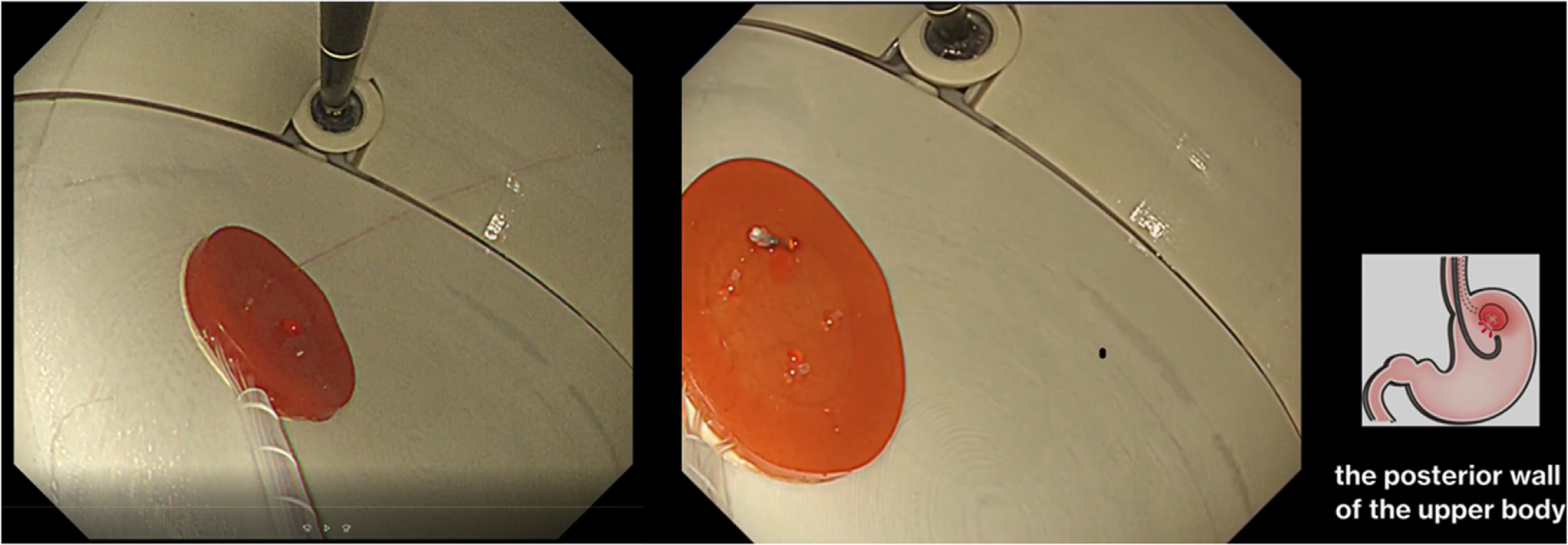
A high-difficulty lesion, such as an ulcer, at the posterior wall of the upper body.

The instructor can also manually stop the bleeding while teaching, depending on the learner’s skill level. In addition, we reproduced the mucosal cauterization reaction by placing a shallow jelly layer on the surface of the ulcer model, resembling the mucus layer (Fig. 8). These products are not currently available commercially but will be available in the future. The approximate prices for reusable stomach lumens are expected to be less than $1,000 USD, and for disposable ulcers are expected to be less than $150 USD per ulcer.

**Figure 8:**
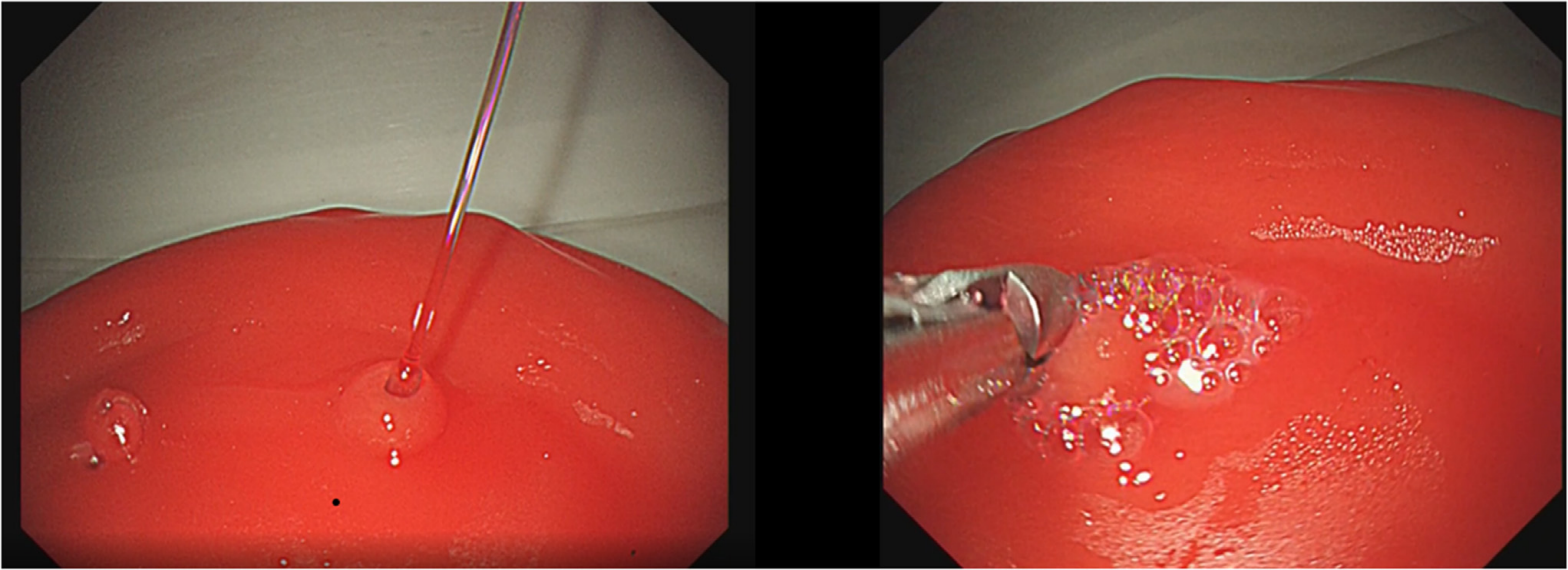
Endoscopic hemostasis by hemostatic grasper producing the mucosal cauterization reaction.

## Limitation

This GI bleeding model may be more challenging than the actual procedure in some locations because it cannot reproduce the deformation of the lumen by controlling air volume in the stomach. It may be more challenging to tightly grasp the clips to stop bleeding in a patient with a fibrotic ulcer bed than in our simulator model, which is made of elastic resin. The lateral hole of the lumen may make it difficult to reproduce a situation with large amounts of bloody intestinal fluid. Future generations of this simulator may need modifications to simulate the difficulty of field-view management in profuse hemorrhage cases.

## Conclusion

A novel GI bleeding simulator for endoscopic hemostasis provides a realistic experience using an actual endoscope and devices while in a calm learning situation without putting the patient at risk. Learners can control the difficulty level by changing the location of the ulcer. We believe this GI bleeding model will be helpful for both beginner and expert endoscopists.

## Disclosure

*This work was supported by* 10.13039/501100001691*Japanese Society for the Promotion of Science KAKENHI* *Grants* *JP17K17591, JP19H03864, and JP22K10460* *. The authors disclosed no financial relationships.*

## Supplementary data

Video 1 Demonstration of novel simulator of endoscopic hemostasis.
